# Therapeutic Efficacy of Intratendinous Delivery of Dexamethasone Using Porous Microspheres for Amelioration of Inflammation and Tendon Degeneration on Achilles Tendinitis in Rats

**DOI:** 10.1155/2020/5052028

**Published:** 2020-01-21

**Authors:** Somang Choi, Mi Hyun Song, Kyu-Sik Shim, Hak-Jun Kim, Youn-Mook Lim, Hae-Ryong Song, Kyeongsoon Park, Sung Eun Kim

**Affiliations:** ^1^Department of Biomedical Science, College of Medicine, Korea University, Anam-dong, Seongbuk-gu, Seoul 02841, Republic of Korea; ^2^Department of Orthopedic Surgery and Rare Diseases Institute, Korea University Medical College, Guro Hospital, #148, Guro-dong, Guro-gu, Seoul 08308, Republic of Korea; ^3^Advanced Radiation Technology Institute, Korea Atomic Energy Research Institute, 1266 Sinjeong-dong, Jeongeup-si, Jeollabuk-do 56212, Republic of Korea; ^4^Ortho-Heal Co., Ltd., #226, Gamasan-ro, Guro-gu, Seoul 08307, Republic of Korea; ^5^Department of Systems Biotechnology, Chung-Ang University, Anseong-si, Gyeonggi-do 17546, Republic of Korea

## Abstract

Achilles tendinitis caused by overuse, aging, or gradual wear induces pain, swelling, and stiffness of Achilles tendon and leads to tendon rupture. This study was performed to investigate the suppression of inflammation responses in interleukin-1*β-* (IL-1*β-*) stimulated tenocytes *in vitro* and the suppression of the progression of Achilles tendinitis-induced rat models *in vivo* using dexamethasone-containing porous microspheres (DEX/PMSs) for a sustained intratendinous DEX delivery. DEX from DEX/PMSs showed the sustained release of DEX. Treatment of IL-1*β*-stimulated tenocytes with DEX/PMSs suppressed the mRNA levels for COX-2, IL-1*β*, IL-6, and TNF-*α*. The intratendinous injection of DEX/PMSs into Achilles tendinitis rats both decreased the mRNA levels for these cytokines and increased mRNA levels for anti-inflammatory cytokines IL-4 and IL-10 in tendon tissues. Furthermore, DEX/PMSs effectively prevented tendon degeneration by enhancing the collagen content and biomechanical properties. Our findings suggest that DEX/PMSs show great potential as a sustained intratendinous delivery system for ameliorating inflammation responses as well as tendon degeneration in Achilles tendinitis.

## 1. Introduction

Tendon disorders caused by overuse, aging, or gradual wear and tear are common in athletes and sedentary peoples. Achilles tendinitis involves chronic pain and swelling [1] and induces an inflammatory response. Many inflammatory markers (e.g., tumor necrosis factor-*α* (TNF-*α*), interleukin-1*β* (IL-1*β*), matrix metalloproteinases (i.e., MMP-2, -3, -9, and -13, among others), and metabolic enzyme (cyclooxygenase-2, COX-2)) are involved in this process [1–3]. Anti-inflammatory treatment of Achilles tendinitis usually entails noninvasive methods such as treating with nonsteroidal anti-inflammatory drugs (NSAIDs) and corticosteroid treatments, ultrasound, and hot and cold compresses [4]. However, the chronic oral administration of NSAIDs is not recommended due to gastrointestinal adverse effects [5].

Glucocorticoids are widely prescribed to reduce inflammatory conditions and to relieve long-term pain [6]. However, despite the strong therapeutic effects, their long-term treatments affect tendon healing deleteriously and induce tendon rupture [7, 8]. Mikolyzk et al. [9] and Torricelli et al. [10] suggested that glucocorticoid treatments reduces tendon strength and also inhibits proliferation and collagen synthesis of tenocytes. Published reports show that the treatment of tendon stem/progenitor cells (TSCs) with DEX reduces TSC proliferation and causes the differentiation of TSCs into nontenocytes [7].

To conquer such drawbacks, drug delivery systems (DDSs) are continuously under development to achieve controlled and/or sustained drug release over a prolonged period of time [11]. DDSs containing liposomes, nanoparticles, 3D scaffolds, and microspheres improve bioavailability and pharmacokinetics [8, 11, 12]. Liposomes, microspheres, and nanoparticles have been used as injectable scaffolds for noninvasive or minimally surgical applications. Injectable scaffolds have some benefits, including brief operation times, little scarring, and increased patient comfort. Porous microspheres (PMSs) with interconnective structures on the surface or interior pores are promising DDSs for the delivery of bioactive drugs, proteins, and cells [13–15].

Herein, we manufactured dexamethasone-containing porous microspheres (DEX/PMSs) using a fluidic device method. We also created an Achilles tendinitis animal model with collagenase treatment. This study was performed to evaluate whether DEX/PMSs have therapeutic responses on the Achilles tendinitis rat model through the intratendinous delivery.

## 2. Materials and Methods

### 2.1. Fabrication of DEX-Contained PMSs (DEX/PMSs)

To produce DEX/PMSs, a simple fluidic device equipped was used as previously described [1, 3, 13]. First, 140 mg of poly (lactic-co-glycolic acid) (PLGA, 50 : 50, Mw: 30,000–60,000 g/mol, Sigma-Aldrich, St. Louis, MO, USA) was clearly solubilized in dichloromethane (DCM, 7 mL). Then, 1.4 mg (1%), 7 mg (5%), and 14 mg (10%) of DEX (Tokyo Chemical Industry Co., Ltd, Tokyo, Japan) were added to PLGA solution with mild shaking for 4 hr, respectively. 750 mg of gelatin (Type A, from porcine skin, Sigma-Aldrich) and 200 mg of poly (vinyl alcohol) (PVA, Mw: 13,000–23,000 g/mol, Sigma-Aldrich) were clearly solubilized in deionized and distilled water (DDW, 10 mL), respectively. Three milliliters of gelatin and 0.5 mL of PVA solution were also added into DEX-contained PLGA solution. The resulting solutions were then homogenized (13,500 rpm, 1 min) to prepare discontinuous phase. As the continuous phase, PVA (1 wt.%) was introduced. DEX/PMSs were fabricated by flowing both discontinuous phase (0.05 mL/min) and continuous phase (2 mL/min). DEX/PMSs were collected and suspended into warm DDW (45°C) for 4 hr to remove the gelatin from the microspheres. After additional washing with DDW, the microspheres were freeze-dried for 3 days. DEX/PMSs prepared at different weights (1.4, 7, and 14 mg) of DEX were designated as DEX (1%)/PMSs, DEX (5%)/PMSs, and DEX (10%)/PMSs, respectively. As a vehicle control with DEX, PMSs alone were also manufactured via the same protocol.

### 2.2. Characterizations of Morphologies and Drug Contents

PMS morphologies with or without DEX were analyzed with SEM (S-2300, Hitachi, Tokyo, Japan). After gold coating, the morphologies of each sample were imaged with SEM. From the microspheres in each group (*n* = 30 pores/sample), average pore sizes of the microspheres were analyzed with the Image J software ((Ver. 1.2, Bethesda, MD, USA). Drug loading contents within DEX/PMSs were calculated using a Flash Multimode Reader (Varioskan™, Thermo Scientific, USA). Briefly, 10 mg of each DEX/PMS was dissolved in 1 mL of DMSO. The DEX loading content within each DEX/PMS group was calculated by analyzing the absorbance at 278 nm.

### 2.3. *In Vitro* Drug Release Profiles

In order to determine the released DEX amount from the DEX/PMSs, 10 mg of each DEX/PMSs was placed in a 15 mL tube containing 1 mL PBS (pH 7.4). Each sample was gently shaken in warm water (37°C) oscillating 100 times/min. At predesignated time schedules, the PBS solution was withdrawn completely and freshly changed with PBS. Released amount of DEX was determined by quantifying the absorbance at 278 nm.

### 2.4. *In Vitro* Cytotoxicity

Cell viability test of each group on tenocytes was indirectly evaluated through the ISO/EN 10993 Part 5 guidelines. The extraction medium was obtained as follows: 10 mg of each microsphere was dispersed in a 1 mL Dulbecco's modified Eagle's medium (DMEM, Gibco BRL, Rockville, MD, USA) contained in 15 mL conical tube, followed by gentle shaking in warm water (37°C) at 100 rpm. 5 × 10^4^ cells of tenocytes were cultured in each well of 96-well plates at 37°C for 24 hr. Then, DMEM was aspirated from each well and extraction medium was treated. After removing the treated extraction medium at predetermined intervals, cell counting kit-8 (CCK-8, Dojindo Molecular Tech., Inc., Tokyo, Japan) reagent was applied to the cells and further incubated at 37°C. After 24 hr, the cell viability was then determined by analyzing the absorbance at 450 nm using a Flash Multimode Reader (Thermo Scientific).

### 2.5. *In Vitro* Evaluation for Anti-Inflammatory Responses of DEX/PMSs in IL-1*β*-Stimulated Tenocytes

To demonstrate whether DEX/PMSs exert anti-inflammatory properties in IL-1*β*-stimulated tenocytes, the mRNA expression levels of cytokines including COX-2, IL-1*β*, IL-6, and TNF-*α* were analyzed with a real-time PCR. Tenocytes (1 × 10^5^ cells/mL/well) were carefully seeded and cultured on each microsphere group (10 mg) in 24-well plates for 24 hr. The final treatment amount of DEX in each group was 1.2 mg for DEX (1%)/PMSs, 6.95 mg for DEX (5%)/PMSs, and 13.8 mg for DEX (10%)/PMSs, respectively. Next, IL-1*β* (100 ng/mL) was added to each group. At predetermined intervals, the cells in each group were collected to isolate total RNA. RNA was extracted from the cells with an RNeasy Plus Mini Kit (Qiagen, Valencia, CA, USA). Then, 1 *μ*g of isolated RNA was reverse-transcribed into cDNA. The primers for proinflammatory cytokines were COX-2, (F) 5′-CAG CCA TAC TAT GCC TCG GA-3′, (R) 5′-GGA TGT CTT GCT CGT CGT TC-3′; IL-1*β*, (F) 5′-CCA CCT CCA GGG ACA GGA TA-3′, (R) 5′-AAC ACG CAG GAC AGG TAC AG -3′; IL-6, (F) 5′-CCG TTT CTA CCT GGA GTT TG-3′, (R) 5′-GTT TGC CGA GTA GAC CTC AT-3′; and TNF-*α*, (F) 5′-CTC CCA GAA AAG CAA GCA AC-3′, (R) 5′-CGA GCA GGA ATG AGA AGA GG-3′. PCR amplification and detection were conducted with an ABI7300 Real-Time Thermal Cycler (Applied Biosystems, Foster, CA, USA). The mRNA levels of these cytokines were normalized to those of GAPDH.

### 2.6. Preparation of Achilles Tendinitis Animal Model and DEX/PMS Treatments

Male Sprague–Dawley rats (8 weeks old) weighing 200 ± 20 g bodyweight were purchased from DooYeol Biotech (Seoul, Korea). All rats were preserved at 22 ± 2°C under a constant 12 h/12 h light and dark exposure cycle and received standard diet pellets (DooYeol Biotech, Seoul, Korea) and water ad libitum. *In vivo* animal studies were approved by the IACUC of Korea University Medical Center (KOREA-2018-0044). Achilles tendinitis rat models were established as follows: under anaesthetization (1% w/v, isoflurane in 2 L oxygen), 50 *μ*L of collagenase Type I (Col (I), 10 mg/mL, Gibco BRL, Rockville, MD, USA) was administered into the right Achilles tendon of each rat using an insulin syringe with a 26G needle. At 1 week, the rats were given 50 *μ*L of carboxymethyl cellulose (CMC) containing each microsphere (10 mg). The actual treated amount of DEX in each group was 60 *μ*g/rat for DEX (1%)/PMSs, 347.5 *μ*g/rat for DEX (5%)/PMSs, and 690 *μ*g/rat for DEX (10%)/PMSs, respectively. At 4 weeks after microsphere injections, the rats were sacrificed for further analysis. Six experimental groups (*n* = 4) are as follows: (I) control (normal), (II) Col (I), (III) Col (I) + PMSs, (IV) Col (I) + DEX (1%)/PMSs, (V) Col (I) + DEX (5%)/PMSs, and (VI) Col (I) + DEX (10%)/PMSs.

### 2.7. Histological Study

The isolated tendon tissues from rats were fixed in 3.7% formaldehyde solution, and dehydrated tissues in ethanol were then embedded in paraffin. The longitudinally sliced tissues (5 *μ*m thickness) were stained with Masson's trichrome to evaluate the reconstruction of collagen fiber.

### 2.8. *In Vivo* Anti-Inflammatory Properties of DEX/PMSs

*In vivo* therapeutic responses of DEX/PMSs on Achilles tendinitis rat models were evaluated by determining the mRNA expression levels of proinflammatory factors (COX-2, IL-1*β*, IL-6, and TNF-*α*) and anti-inflammatory factors (IL-4 and IL-10) in tendons treated with each sample using real-time PCR. At 4 weeks after each sample treatment, the isolated and frozen tendon tissues (5 mg) were homogenized using TRIzol reagent (Life Technologies, Carlsbad, CA, USA). The total RNA extraction was done from the homogenized tissues using the RNeasy Mini Kit (Qiagen). The total RNA (1 *μ*g) was reverse-transcribed into cDNA using AccuPower RT PreMix (Bioneer). The primers for the proinflammatory cytokines were the same as those used in *in vitro* anti-inflammatory study. On the other hand, primers for two anti-inflammatory cytokines IL-4 and IL-10 were IL-4, (F) 5′-ACA GGA GAA GGG ACG CCA T-3′, (R) 5′-GAA GCC CTA CAG ACG AGC TCA-3′; and IL-10, (F) 5′-GGT TGC CAA GCC TTA TCG GA-3′, (R) 5′-ACC TGC TCC ACT GCC TTG CT-3′. PCR amplification and detection were performed in the same manner described above for analyzing mRNA levels *in vitro*.

### 2.9. Hydroxyproline Assay

For hydroxyproline assay, 5 mg of Achilles tendon tissues were hydrolyzed by using 6 N HCl at 120°C for 12 hr, and the hydrolyzed tissues were neutralized with NaOH. After 6 *μ*L of chloramine T (60 mM) solution was mixed with oxidation buffer (94 *μ*L), this solution was added to each sample or standard solution. The transferred resulting solution into a 96-well plate was incubated for 20 min. Next, 100 *μ*L of *p*-dimethylaminobenzaldehyde was added to each well, and then further incubated at 60°C for 90 min. Finally, the absorbance was recorded at 450 nm using a Flash Multimode Reader.

### 2.10. Biomechanical Tests

After tendon specimens were carefully fixed to a specially designed device, their biomechanical properties were tested with an Instron Mechanical Tester (AG-10KNX, Shimadzu, Japan) at a cross-head speed of 5 mm/min with a preload force (1 N). The ultimate tensile strength and stiffness were obtained as maximum stress or force per unit area and force required per unit displacement, respectively.

### 2.11. Statistical Analysis

Data are expressed as mean ± SD (*n* = 4). Statistical comparisons were done via one-way analysis of variance (ANOVA) using Systat software (Chicago, IL, USA). *P* values less than 0.05 or 0.01 were statistically significant.

## 3. Results

### 3.1. Characterizations of PMSs and DEX/PMSs

**Figure 1:**
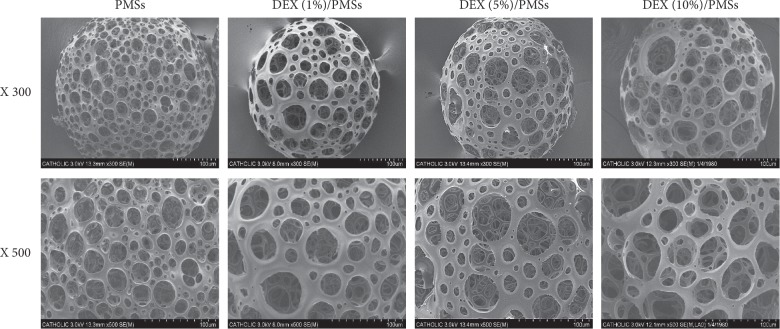
Scanning electron microscopic (SEM) images of PMSs, DEX (1%)/PMSs, DEX (5%)/PMSs, and DEX (10%)/PMSs.

The morphologies of manufactured PMSs or DEX/PMSs were analyzed with SEM. All microspheres including PMSs and three kinds of DEX/PMSs were spherical types and displayed highly porous structures and similar pore sizes (Figure 1). The pore sizes were 25.08 ± 6.12 *μ*m for PMSs, 25.52 ± 4.32 *μ*m for DEX (1%)/PMSs, 24.72 ± 7.29 *μ*m for DEX (5%)/PMSs, and 24.72 ± 7.29 *μ*m for DEX (10%)/PMSs, respectively. Three DEX/PMSs such as DEX (1%)/PMSs, DEX (5%)/PMSs, and DEX (10%)/PMSs contained 1.27 ± 0.02 *μ*g, 6.95 ± 0.01 *μ*g, and 13.75 ± 0.26 *μ*g per 10 mg of PMSs, respectively.

### 3.2. *In Vitro* Cytotoxicity and DEX Release

**Figure 2:**
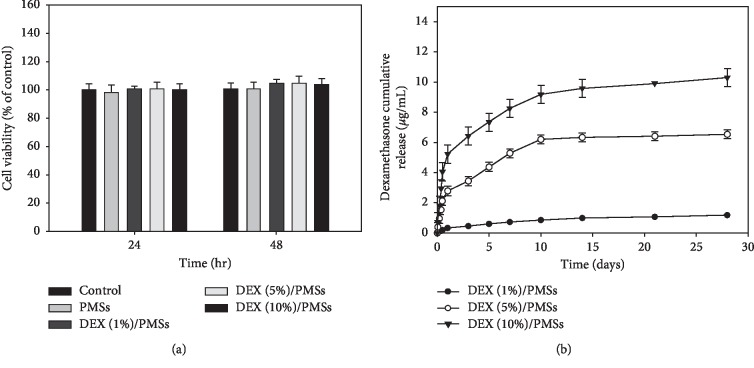
(a) Cytotoxicity of PMSs, DEX (1%)/PMSs, DEX (5%)/PMSs, and DEX (10%)/PMSs at 24 and 48 hr. (b) Cumulative dexamethasone release from DEX (1%)/PMSs, DEX (5%)/PMSs, and DEX (10%)/PMSs. The error bars are presented as the standard deviation of measurements for 10 mg PMSs with or without DEX in each group (*n* = 4).

Cytotoxicities of PMSs and three DEX/PMSs were evaluated against tenocytes. As shown in Figure 2(a), cell viabilities were maintained at almost 95% compared to the control group, indicating that these microspheres are nontoxic.  Figure 2(b) displays the *in vitro* release of DEX from three types of DEX/PMSs. At day 1, there was 0.32 ± 0.00 *μ*g of DEX released from DEX (1%)/PMSs, 2.77 ± 0.30 *μ*g from DEX (5%)/PMSs, and 5.23 ± 0.60 *μ*g from DEX (10%)/PMSs, respectively. During 28 days, the released DEX from three DEX/PMSs such as DEX (1%)/PMSs, DEX (5%)/PMSs, and DEX (10%)/PMSs was 1.18 ± 0.01 *μ*g, 6.53 ± 0.29 *μ*g, and 10.30 ± 0.59 *μ*g, respectively.

### 3.3. *In Vitro* Anti-Inflammatory Responses by Confirming mRNA Expression Levels for Proinflammatory Cytokines

**Figure 3:**
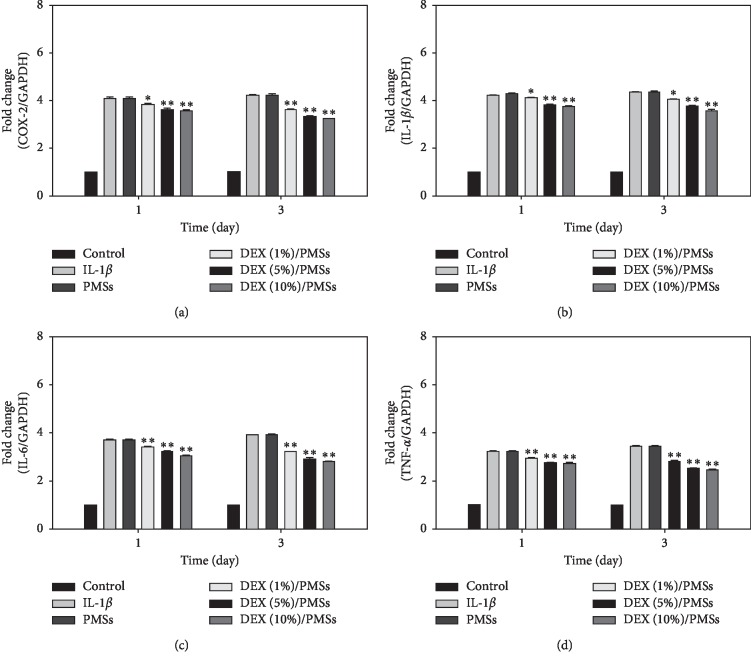
Relative mRNA levels of proinflammatory cytokines including (a) COX-2, (b) IL-1*β*, (c) IL-6, and (d) TNF-*α* in IL-1*β*-stimulated tenocytes on days 1 and 3. The error bars are presented as the standard deviation of measurements for 10 mg PMSs with or without DEX in each group (*n* = 4). ^*∗*^*P* < 0.05 and ^*∗∗*^*P* < 0.01 as compared to PMS group.

The mRNA levels of four kinds of cytokines (i.e., COX-2, IL-1*β*, IL-6, and TNF-*α*) in IL-1*β*-stimulated tenocytes grown on PMSs and three kinds of DEX/PMSs were shown in Figure 3. IL-1*β*-stimulated cells only and PMSs controls displayed the greatest mRNA expression of four cytokines. However, their mRNA levels in cells grown on DEX/PMSs dose-dependently decreased compared with those of PMSs alone on days 1 and 3. Moreover, among three DEX/PMS groups, DEX (10%)/PMSs showed slightly lower mRNA levels of these four cytokines.

### 3.4. Histological Evaluation of Achilles Tendon Tissues

**Figure 4:**
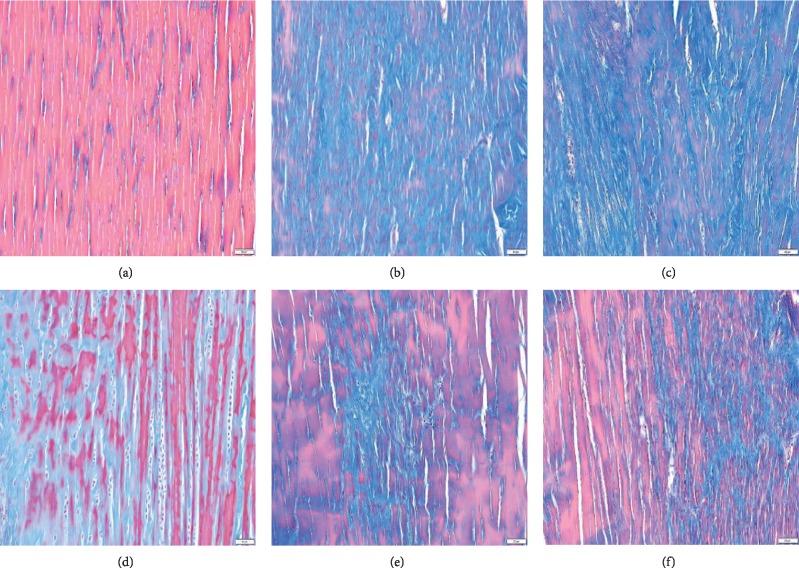
Masson's trichrome staining at 5 weeks after collagenase (Col (I)) injection into tendon tissues and 4 weeks after treatment with PMSs, DEX (1%)/PMSs, DEX (5%)/PMSs, and DEX (10%)/PMSs. Groups are divided as follows: (a) control (no treatment); (b) Col (I); (c) Col (I) + PMSs; (d) Col (I) + DEX (1%)/PMSs; (e) Col (I) + DEX (5%)/PMSs; (f) Col (I) + DEX (10%)/PMSs. Scale bar: 100 *µ*m. Red: collagen fibers; blue: collagen matrix collapse.

To confirm the suppression of Achilles tendinitis progression, the tendon tissues were stained with Masson's trichrome staining at 4 weeks after PMSs and three DEX/PMSs injections (Figures 4(a)–4(f)). Collagenase and PMS-treated groups showed collagen fiber disruption and no aligned collagen fibers (Figures 4(b) and 4(c)). Groups injected with three types of DEX/PMSs gradually decreased collagen fiber breakdown in a dose-dependent fashion (Figures 4(d)–4(f)). Among three DEX/PMSs treatment groups, DEX (10%)/PMSs displayed more therapeutic effects than others.

### 3.5. *In Vivo* Inhibition of Inflammation Responses of DEX/PMSs in Achilles Tendinitis Models

**Figure 5:**
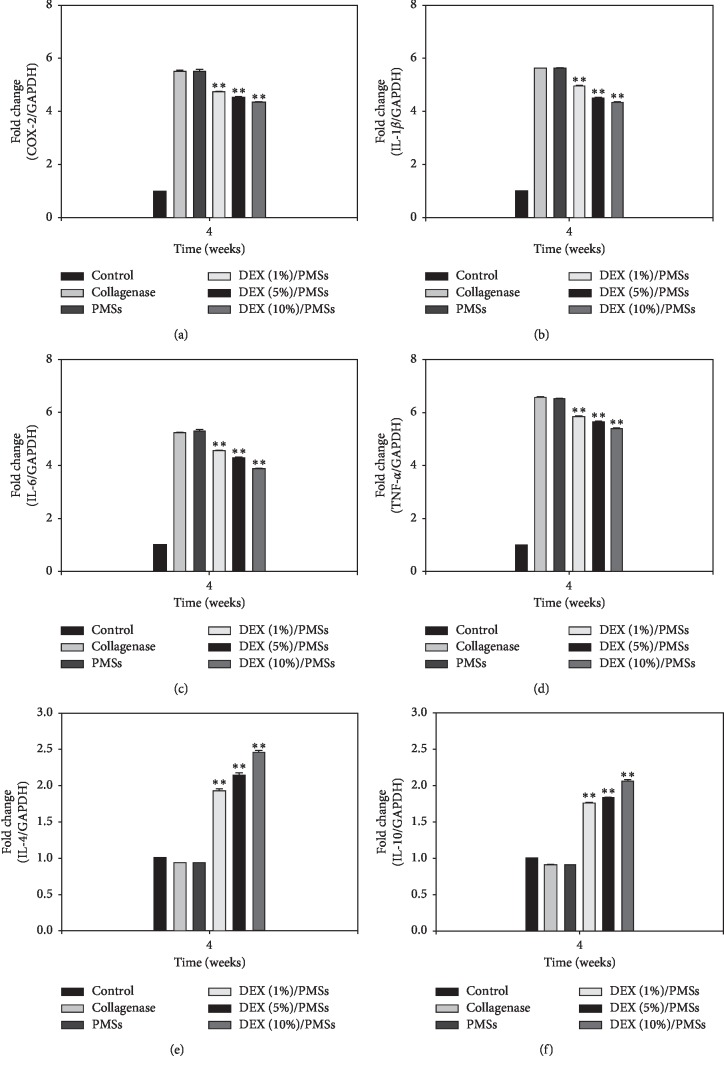
Relative mRNA levels of proinflammatory cytokines including (a) COX-2, (b) IL-1*β*, (c) IL-6, (d) TNF-*α*, (e) IL-4, and (f) IL-10 in tendon tissues at 4 weeks after treatment by group. The error bars are presented as the standard deviation of measurements in Achilles tendon tissues treated with collagenase and PMSs with or without DEX in each group (*n* = 4). ^*∗∗*^*P* < 0.01 as compared to PMS group.

To demonstrate *in vivo* inhibition of inflammation responses of DEX/PMSs in Achilles tendinitis models, the mRNA levels for pro- and anti-inflammatory cytokines from tendon tissues were measured using the real-time PCR. Figures 5(a)–5(d) show that the mRNA levels of proinflammatory cytokines in Col (I)- and PMSs-injected groups were greatly upregulated compared to those in PMSs containing DEX. Moreover, significant differences in mRNA levels for four kinds of four proinflammatory factors COX-2, IL-1*β*, IL-6, and TNF-*α* were observed among the DEX/PMS-treated groups. Consistently with *in vitro* study, the DEX (10%)/PMS-treated group showed much lower mRNA levels of these cytokines than the two other DEX/PMSs. In contrast, DEX/PMSs treatments remarkably elevated the mRNA expressions of IL-4 and IL-10 in a dose-dependent response (Figures 5(e) and 5(f)).

### 3.6. *In Vivo* Preventive Effects of Tendon Degeneration

**Figure 6:**
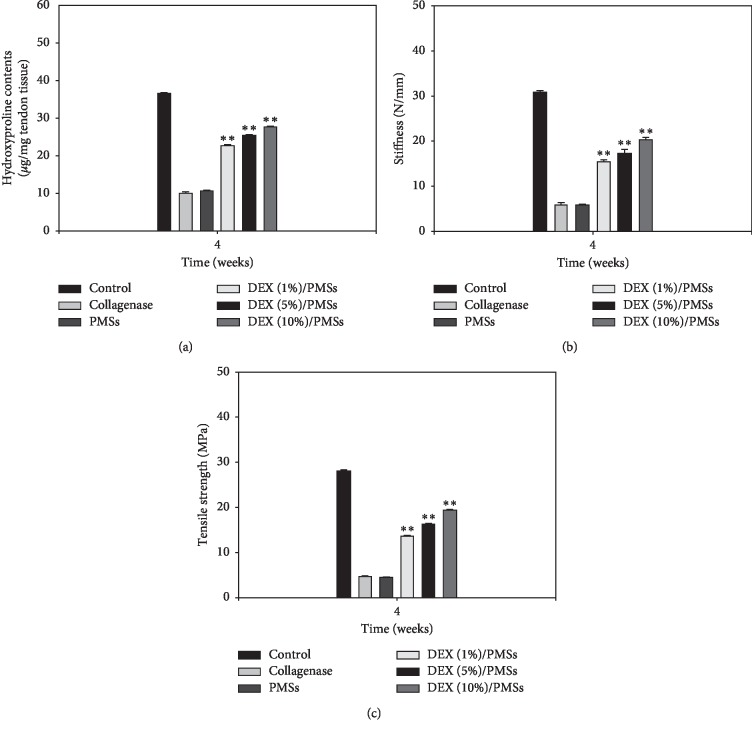
(a) Hydroxyproline content, (b) stiffness, and (c) tensile strength of tendon tissues in collagenase-induced Achilles tendinitis rat models at 4 weeks after treatment by group. The error bars are presented as the standard deviation of measurements in Achilles tendon tissues treated with collagenase and PMSs with or without DEX in each group (*n* = 4). ^*∗∗*^*P* < 0.01 as compared to PMS group.

To demonstrate the *in vivo* preventive effects of tendon degeneration, hydroxyproline contents were measured at 4 weeks after treatments (Figure 6(a)). Significant differences in hydroxyproline content were not detected between Col (I)- and PMS-treated groups. In contrast, DEX/PMS-treated groups dose-dependently increased the hydroxyproline content. To further confirm the preventive effects of tendon degeneration by DEX/PMSs, we conducted biomechanical properties on Achilles tendon tissues (Figures 6(b) and 6(c)). In Col (I)- and PMS-treated groups, the values of the stiffness and tensile strength were much lower compared to those in the control group. In contrast, three DEX/PMSs treatments gradually and dose-dependently increased these values compared to Col (I)- and PMS-treated groups.

## 4. Discussion

Achilles tendinopathy usually involves Achilles tendinosis, Achilles tendinitis, and paratendinitis. Among them, Achilles tendinitis is associated with inflammation via acute trauma and chronic overuse. Glucocorticoid treatment via injection plays a protective role by suppressing inflammation. However, several adverse effects including pain after drug injection, rupture of the Achilles tendon, skin atrophy, and depigmentation are induced by repetitive injections [7, 8, 16]. For these reasons, we examined whether DEX/PMSs suppressed inflammatory responses and effectively prevented tendon degeneration in Achilles tendinitis models via the intratendinous treatment of DEX/PMSs.

DEX is a synthetic glucocorticoid that ameliorates inflammation and pain, such as tendinopathy with chronic inflammation and degeneration of tendons [6, 7]. It is about 5 to 10 times more potent than prednisolone and has a half-life of about 36–72 h [17, 18]. DEX has anti-inflammatory efficacy by blocking mesylate and actinomycin D on leukocyte infiltration in the inflamed sites [18]. However, oral administration of DEX for a long period induces detrimental effects such as gastrointestinal bleeding, ulceration, and perforation. Due to these drawbacks, porous microspheres (PMSs) as a drug delivery system were used to locally deliver DEX into the Achilles tendon to suppress inflammation. PMSs were fabricated with a simple fluidic device composed of both discontinuous and continuous channels [3, 13, 19, 20]. The fluidic device allows easy control of pore size, porosity, and the size of microspheres. SEM images exhibited that the manufacture PMSs and DEX/PMSs are both spherical in shape with high porosity and interconnected pores. These results were consistent with SEM results from our earlier studies [3, 13, 20]. For the *in vitro* drug release study, the initial fast release of DEX is due to the diffusion of DEX on the surface of microspheres. After that, DEX release from the microspheres was incomplete during the 4 weeks when considering polymer degradation, suggesting the suitability of DEX/PMSs as a sustainable drug release vehicle.

As fibroblast-like differentiated cells, tenocytes were found throughout the tendon tissues. Moreover, they produce an extracellular matrix (ECM) and early collagen fiber assembly. In the present study, tenocytes were treated with IL-1*β* in order to mimic the inflamed condition *in vitro*. Our previous studies reported that LPS-stimulated tenocytes increase gene expression levels for cytokines (i.e., ADAMTS-5, COX-2, IL-6, TNF-*α*) as well as ECM-degrading proteinases (i.e., MMP-3 and MMP-13) [1, 3]. We found that DEX/PMS systems markedly suppressed the mRNA levels of these cytokines in IL-1*β*-stimulated tenocytes. Previous studies have shown that cells treated by DEX suppress the expression of several cytokines such as IL-6, IL-8, granulocyte-macrophage colony-stimulating factor (GM-CSF), regulated on activation normal T cell expressed and secreted (RANTES), and TNF-*α* [21–24]. Moreover, our results are consistent with earlier findings that DEX inhibits various inflammatory cytokines IL-1*β*, IL-2, IL-6, IL-8, and TNF-*α* genes in LPS-induced A549 cells in a dose-dependent fashion [24]. Overall, these data indicate that DEX/PMSs have potent anti-inflammatory properties by effectively inhibiting proinflammatory cytokine mRNA levels in IL-1*β*-stimulated tenocytes.

Achilles tendinitis animal models are generally induced by collagenase injection because it induces not only an inflammatory response in tendons but also reproducible tendon degeneration. Also, the injected collagenase induces the disruption of collagen fibrils in tendon tissues and changes the biochemical factors and biomechanical characteristics of the tendon, more closely resembling the histopathologic symptoms and dysfunctions of human tendinopathy [25–27]. Thus, rats with Achilles tendon breakdown by Col (I) injection were used to investigate both *in vivo* inhibition of inflammation and prevention of tendon degeneration for the DEX/PMSs system. Histological data demonstrated that normal tendon tissues had well-organized collagen fiber and no collagen breakdown was found, whereas Col (I)-treated tendons displayed tangled collagen fibers and dramatic collagen matrix breakdown. PMS-treated tendons did not preserve the collagen matrix breakdown. Meanwhile, DEX/PMS-treated tendons exhibited the restoration of the collagen matrix organization in a dose-dependent response. These data suggest that locally injected DEX/PMSs contributed to tendon restoration against the progression of Achilles tendinitis.

Healing of tendon disorders such as rupture or tendinopathy normally occurs in three phases: inflammation, proliferation or collagen-production, and remodeling [28, 29]. During the healing progression of tendon tendinopathy, multiple cytokines play an important role in promoting healing [30–32]. Indeed, whereas anti-inflammatory cytokines attract fibroblasts to the site of restoration, excessive inflammatory responses result in unsatisfactory clinical outcomes [33, 34]. Macrophages involved in the phagocytosis of necrotic debris play a pivotal role in promoting fibroblast proliferation and tissue repair by inducing ECM modification through the release of chemotaxis and growth factors [33, 34]. Macrophages are categorized into two groups: classically activated proinflammatory (i.e., IL-1*β*, IL-6, and MMPs) macrophages (M1) and alternatively activated anti-inflammatory (e.g., IL-4, IL-10, and IL-13) macrophages (M2) [33, 34]. Classically activated M1 macrophages are involved in accelerating ECM disruption, inflammation, and apoptosis, whereas alternatively activated M2 macrophages contribute to the coordination of anti-inflammatory functions and ECM deposition as well as advanced tissue repair and remodeling [33–36].

To investigate the therapeutic effect of DEX/PMSs on Achilles tendinitis models, we measured mRNA expression of pro- and anti-inflammatory cytokines in tendon tissues. The mRNA levels of cytokines COX-2, IL-1*β*, IL-6, and TNF-*α* were significantly increased and those of cytokines IL-4 and IL-10 were not increased in the PMS-treated groups, indicating that microspheres without drug had no anti-inflammatory effects. In contrast, DEX/PMSs treatments not only downregulated proinflammatory cytokines but also upregulated the mRNA levels of IL-4 and IL-10 in a dose-dependent fashion. The strongest differences were observed on DEX (10%)/PMSs relative to the other DEX/PMSs-treated groups, implying a superior therapeutic effect.

In order to demonstrate *in vivo* tendon amelioration via DEX/PMS injection, we used a hydroxyproline assay and biomechanical studies. According to reports, hydroxyproline is a specific indicator of collagen content, an extracellular component in connective tissues (e.g., skin, tendon, cartilage, and bone), as well as an important regulator of multiple biochemical and physiological processes [37–39]. The hydroxyproline content of treated tendon tissues was greatly increased in a dose-dependent response after DEX/PMS injections compared with PMS injection alone. Moreover, tendon tissues treated with DEX (10%)/PMSs showed significantly improved tissue quality compared with PMSs, DEX (1%)/PMSs, and DEX (1%)/PMSs, as supported by biomechanical studies. Overall, this study demonstrates that the long-term delivery system of DEX/PMSs can exert anti-inflammatory effects and promote tendon amelioration, as shown in a Col (I)-induced Achilles tendinitis rat model.

## 5. Conclusion

Here, we manufactured DEX/PMSs for long-term and sustainable DEX delivery. The DEX/PMSs clearly suppressed the mRNA levels of cytokines COX-2, IL-1*β*, IL-6, and TNF-*α in vitro* in IL-1*β*-stimulated tenocytes. In agreement, *in vivo* results demonstrated that DEX/PMSs not only significantly suppressed mRNA levels of four kinds of proinflammatory cytokines but also greatly enhanced mRNA levels of anti-inflammatory cytokines IL-4 and IL-10 in collagenase-induced tendon tissue. DEX/PMSs further provided improved tendon amelioration by showing the increase of collagen content, stiffness, and tensile strength. Overall, this study demonstrates that the sustained local delivery of DEX using PMSs shows promise as a therapeutic injectable material to ameliorate inflammation and Achilles tendinitis.

## Data Availability

The data used to support the results of this study are available from the corresponding author upon request.
